# Curcumin Rescues Doxorubicin Responsiveness via Regulating Aurora a Signaling Network in Breast Cancer Cells

**DOI:** 10.31557/APJCP.2021.22.3.957

**Published:** 2021-03

**Authors:** Souvick Biswas, Elizabeth Mahapatra, Archismaan Ghosh, Salini Das, Madhumita Roy, Sutapa Mukherjee

**Affiliations:** *Department of Environmental Carcinogenesis & Toxicology, Chittaranjan National Cancer Institute, 37, S. P. Mukherjee Road, Kolkata, India. *

**Keywords:** Aurora A, doxorubicin insensitivity, breast cancer, curcumin

## Abstract

**Background:**

Insensitivity towards anthracycline drugs like doxorubicin poses a significant challenge in the treatment of breast cancer. Among several factors, Aurora A (a mitotic serine threonine kinase) plays crucial roles in acquiring non-responsiveness towards doxorubicin. However, the mechanisms underlying need to be elucidated. The present study was therefore designed to evaluate the underlying mechanisms of Aurora A mediated doxorubicin insensitivity in MCF-7Dox/R, an isolated resistant-subline of MCF-7 (breast adenocarcinoma cell line). Effect of curcumin, a natural phytochemical in restoring doxorubicin sensitivity by targeting Aurora A was assessed furthermore.

**Methods:**

A doxorubicin resistant subline (MCF-7Dox/R) was isolated from the parental MCF-7 cells by treating the cell with gradual step-wise increasing concentration of the drug. Expressions of Aurora A and its target proteins (Akt, IκBα and NFκB) were assessed in both parental and MCF-7Dox/R cells. Both the cell lines were pretreated with curcumin prior to doxorubicin treatment. Cellular proliferation rate was measured using BrdU (5-bromo-2’-deoxyuridine) assay kit. Intracellular doxorubicin accumulation was estimated spectrofluorimetrically. Cellular uptake of curcumin (spectrophotometric and spectrofluorimetric method) and its nuclear localization was confirmed by confocal microscopic study. Protein expressions were determined by western blot analysis. Localization of Aurora A was ascertained by immunofluorescence assay. To explore the possible outcome of impact of curcumin on Aurora A, cell-cycle distribution and apoptosis were performed subsequently.

**Results:**

Higher expressions of Aurora A in MCF-7Dox/R cells led to phosphorylation of Akt as well as IκBα. Phosphorylated IκBα preceded release of NFκB. Phospho-Akt, NFκB consequentially decreased doxorubicin accumulation by enhancing the expressions of ABCG2 and Pgp1 respectively. Curcumin by regulating Aurora A and its target molecules sensitized resistant subline towards doxorubicin mediated G2/M-arrest and apoptosis.

**Conclusion:**

Molecular targeting of Aurora A by curcumin restores chemosensitivity by increasing the efficacy of doxorubicin in breast cancer.

## Introduction

Doxorubicin, conventionally used as chemotherapeutic agent against breast cancer limits its therapeutic benefits due to development of acquired insensitivity following long-term therapy (Christowitz et al., 2019). Therefore, understanding the molecular factors hindering the responsiveness of breast cancer cells towards doxorubicin is crucial to unravel and prevent therapeutic insensitivity. 

Human Aurora A protein belonging to the family of serine/threonine kinases is expressed within the centrosome during the early S phase and helps in centrosome duplication and maturation. During G2/M transition Aurora A facilitates mitotic entry by triggering duplicated centrosomes to be separated (Tang et al., 2017). Aurora A undergoes autophosphorylation at Threonine 288 residue to become functionally active. In a wide variety of cancers including breast cancer, aberrant expressions or gene amplification of Aurora A has been documented; indicating its involvement in prosurvival activity and tumorigenesis (D’Assoro et al., 2013; Ferchichi et al., 2013; Zardavas et al., 2014; Cirak et al., 2015). Emerging evidences have documented an association between Aurora A overexpression and decreased chemosensitivity in cancer (He et al., 2014; Kuang et al., 2017; Wang et al., 2017). In triple negative breast cancer cells, response towards doxorubicin was reported to be restored by administration of Aurora A inhibitor (Romanelli et al., 2012). Apart from mitotic involvement, aberrant activation of the non-canonical Aurora A/SMAD5 oncogenic axis in breast cancer has been documented, which eventually led to chemoinsensitivity (Opyrchal et al., 2017). However, the mechanism underlying chemoinsensitivity by Aurora A is yet to be elucidated.

Plant derived natural compounds like curcumin are pharmacologically safe and cost effective having anticancer properties with potential chemosensitizing efficacy (Roy et al., 2011; Vinod et al., 2014; Turrini et al., 2014).

Based on these literature surveys, we felt curious to look into the underlying molecular mechanisms behind doxorubicin insensitivity due to Aurora A. Aurora A downstream Signaling bestowed with acquirement of chemo-insensitivity was explored furthermore. Restoration of sensitivity of breast cancer cells towards doxorubicin by plant derived natural compound curcumin was evaluated as well. 

## Materials and Methods

*Chemicals*

The primary antibodies used for the study anti-Pgp1, anti-ABCG2, anti-phospho-p53(S315), anti-phospho-Akt(S473), anti-phospho- IκBα(S32), anti- NFκB p65, anti-TBP and FITC-conjugated anti-rabbit IgG were purchased from Genetex, CA, USA. Aurora A antibody, Phospho-Aurora A (T288) antibody, β-actin and p21 antibodies were procured from abcam, Cambridge, UK. BrdU (5-bromo-2’-deoxyuridine) assay kit was purchased from Calbiochem, USA. MTT (3-(4,5-Dimethylthiazol-2-yl)-2,5-Diphenyltetrazolium Bromide), BSA (Bovine Serum Albumin), PI (Propidium Iodide), DAPI (4′,6-diamidino-2-phenylindole), Aurora-A Inhibitor I, Akt1/2 kinase inhibitor and curcumin were purchased from Sigma-Aldrich, USA. Annexin V FITC Assay Kit was purchased from Cayman Chemical, MI, USA. Doxorubicin used in the study was obtained Pfizer Inc, USA. 

*Isolation of cell line*

Human breast adenocarcinoma cell line (MCF-7) was purchased from National Centre for Cell Science, Pune, India. A resistant subline was derived from parental MCF-7 by treating exponentially growing cells with stepwise increasing concentration of doxorubicin with an initial dose of 1 nM. Cells were maintained in each concentration for at least 6 passages and finally a colony was isolated at 500nM. The isolated resistant clone was designated as MCF-7Dox/R. Before experimentation, MCF-7Dox/R cells were maintained in drug free medium for at least one week. 

*Maintenance of cell lines*

MCF-7 and MCF-7Dox/R cells were maintained in Minimum Essential Medium Eagle (MEM) supplemented with 10% heat inactivated Fetal Bovine Serum (FBS) and antibiotics. Cells were maintained at 370C in a humidified CO_2_ incubator having 5% CO_2 _/95% air.

*Cellular proliferation assay*

BrdU (5-bromo-2’-deoxyuridine) cell proliferation assay kit (Calbiochem) was used for measuring proliferation rate following the instructions provided in the kit with some adaptations of the protocol of Yang et al., 2010. Briefly, cells at a density of 1x105 cells/ml were seeded into a 96-well culture plate. After 24h, cells were incubated with BrdU working solution for additional 12h. Supernatant was removed thereafter followed by fixation and denaturation with the Fixative/Denaturing Solution for 30 min at room temperature (RT). Anti-BrdU antibody solution (100 µl) was added and allowed to incubate for 1h at RT. Secondary antibody solution was added after washing of unbound primary antibody followed by incubation for 30 min. Finally, substrate solution was added and incubated in the dark for 15 min for terminating the reaction before measuring the amount of incorporated BrdU using Spectrophotometric plate reader at dual wavelengths of 450-540 nm. 

*Doxorubicin accumulation study*

Intracellular accumulation study was carried out in both parental and resistant cells following the protocol of Al-Malky et al., 2020, with some modifications. MCF-7 and MCF-7Dox/R cells (1x105 cells/well) were seeded in 24-well plate and allowed to grow overnight. Exponentially growing cells were treated with 5 μM doxorubicin for 2h. Thereafter cells were harvested, centrifuged, washed thrice in cold PBS to wash out excess drug. Fluorescence intensity was recorded at an excitation (480 nm) and emission (550 nm) using Spectrofluorimeter. 

*MTT assay*

MTT assay was performed according to the standard laboratory protocol (Roy et al., 2015). Confluent cells (MCF-7 and MCF-7Dox/R) were pre-treated with curcumin (25 µM) followed by logarithmic doses of doxorubicin treatment for 24h. Each well was then added with MTT solution (1.2 mg/ml in water) and kept for incubation for 5h. Thereafter, ELISA plate was centrifuged, DMSO was added to dissolve MTT-formazan product (purple colored) and estimated by measuring absorbance at 570 nm in an ELISA plate reader.

*Western blotting*

Western blot analysis was performed using the whole cell lysates. Briefly, treated cells were harvested, washed in wash buffer and lysed in lysis buffer. Lysates were centrifuged and protein concentrations were quantified following Lowry’s method. Equally loaded proteins from cell lysates were then electrophoresed on SDS-polyacrylamide gel using electrophoresis buffer (Tris: 25 mM, glycine: 192 mM, SDS: 20%) and separated proteins were electro-transferred to nitrocellulose membranes using transfer buffer (Tris: 250 mM, glycine: 192 mM, methanol: 10%) and blocked in 5% BSA. The membranes were then incubated with primary antibodies to react overnight at 40C; thereafter washed with TBST (Tris Buffered Saline with Tween20) followed by treatment with alkaline phosphatase conjugated anti-mouse IgG or anti-rabbit IgG (1:1000 dilutions in TBS) depending on the specificity of primary antibodies. Membranes were finally treated with BCIP/NBT to visualise the proteins. For determining the expression of NFκB p65 subunit, p21 and TBP; nuclear proteins were isolated following the standard laboratory method (Roy et al., 2011), followed by western blotting as mentioned above. 

*Semi-quantitative reverse transcription PCR analysis (RT-PCR)*

Isolation of total cellular RNA was performed using RNAqueous 4PCR kit (Ambion/ Applied Biosystem) according to manufacturer’s instructions. cDNA was synthesized from 2 μg of total RNA using RetroScript kit (Ambion/Applied Biosystem). The cDNA was amplified by PCR using forward and reverse primer sequences of Aurora A (Forward primer 5’-AATTGCAGATTTTGGGTGGT-3’; Reverse primer 5’-AAACTTCAGTAGCATGTTCCTGTC-3’). *β-actin/ACTB* gene (Forward primer 5’-CTGGAACGGTGAAGGTGACA-3’; Reverse primer 5’-AAGGGACTTCCTGTAACAACGCA-3’) was used as loading control. PCR product was analyzed by electrophoresis on 2% agarose gel and visualized by staining with ethidium bromide (EtBr) under Gel Documentation System according to the laboratory protocol (Roy et al, 2015). 

*Spectrophotometric and spectrofluorimetric assay for determining intracellular curcumin uptake*

Cells (1.5 x10^5^ cells/tissue culture plate) were seeded and confluent cells were treated with curcumin for different time intervals (0, 1, 2, 4, 8h) and thereafter harvested, centrifuged, washed thrice in cold PBS to wash out excess curcumin. Methanol was added to air dried pellets, resuspended and sonicated until the methanolic lysate containing curcumin was prepared. Curcumin loaded methanolic lysate was centrifuged and supernatant was taken to record data using both spectrophotometer and spectrofluorimeter at an excitation/emission range of curcumin 420 nm/530 nm respectively for MCF-7 and MCF-7Dox/R cells following the protocol of Kunwar et al., (2008).

*Localization of curcumin study by confocal microscopy*

Cells seeded in coverslips, placed in a 6 well confocal plate were treated with curcumin for different time periods and finally processed for fixation using 2% paraformaldehyde in PBS followed by washing and addition of 1% Triton X solution. Cover slips were stained with DAPI (1 μg/ml) according to the method of Kunwar et al, 2008. Excess stain was washed with PBS, air dried and mounted with glycerol. Fluorescence imaging of cells were performed with an Olympus Fluoview-500 confocal laser-scanning microscope (Olympus, Tokyo, Japan) equipped with a multi-Argon laser for excitation at 458, 488 and 515 nm.

*Immunofluorescence study of Aurora A*

Confluent cells seeded on cover slips and placed in 6 well culture plates were treated with curcumin for 6h. After proper rinsing and fixing with 2% paraformaldehyde solution, cells were subjected to permeabilization with 0.1% Triton X-100 for 15-20 mins at RT. Plates were blocked in 2% BSA for 2h, fixed and blocked cells were stained with primary antibody (according to the protocol of Vantangoli et al., (2015) with slight modifications) against Aurora A (diluted in PBB; 0.5% BSA in PBS) solution. Cover-slips were washed in PBST (PBS+0.1% Tween20) for 5 times followed by the addition of FITC conjugated secondary antibody (2h, RT). Cells were stained with DAPI and mounted on microscope slides using DPX. Immunostained cells were then visualized using fluorescent microscope.

*Cell cycle analysis*

Treated cells were harvested, counted and equal number of cells (2x10^6^) taken for each experimentation were washed with cold PBS followed by fixation with 70% chilled ethanol according to the standard laboratory protocol (Sarkar et al, 2013). Proper fixation was done in ice for 30 min and centrifuged for 5 min at 40C to remove residual ethanol. Cell pellets were suspended in 1 ml DNA binding solution containing 200 μg/ml RNase A and 50 μg/ml PI and incubated in dark for 30 min. Finally cells were analysed using a FACScan flow cytometer (Beckton Dickinson), and CellQuest software. Fluorescence was captured for each determination on FL2H channel with logarithmic amplification by counting 10,000 cells. 

*Detection of apoptosis by AnnexinV-FITC/PI staining*

Cells (3x10^5^) were seeded, allowed to grow and exponentially growing cells were treated with curcumin for detection of apoptosis following the instructions provided with the kit. Treated cells were harvested, and binding buffer was added (1X), centrifuged and 50 μl AnnexinV-FITC/PI staining solution was added; incubated for 10 min (RT) in the dark. Suspension was again centrifuged at 400xg at RT to remove excess stain followed by addition of 1X binding buffer. Stained cells were then observed under fluorescence microscope having excitation and emission maxima at 535 nm and 617 nm respectively.

*Morphological examination of cells by PI*

Treated cells were harvested, washed with PBS and centrifuged and to the pellet, PI was added (final: 50 µg/ml) as per the standard laboratory method (Sarkar et al, 2013). Cells were then incubated in the dark at RT for 10 min. Treated cells along with control one was spread over slides, covered with cover slips, and examined under the fluorescent microscope (excitation and emission spectra of PI is 496 nm and 636 nm) Number of apoptotic and normal cells were counted in each slide. 

*Statistical analysis*

GraphPad Prism statistical program was used for statistical analysis and student t test was performed. Differences among means are considered statistically significant when the p-value is less than 0.005. 

## Results

*Aurora A is overexpressed in MCF-7Dox/R compared to parental MCF-7 and increases the proliferative rate of MCF-7Dox/R*

**Figure 1:**
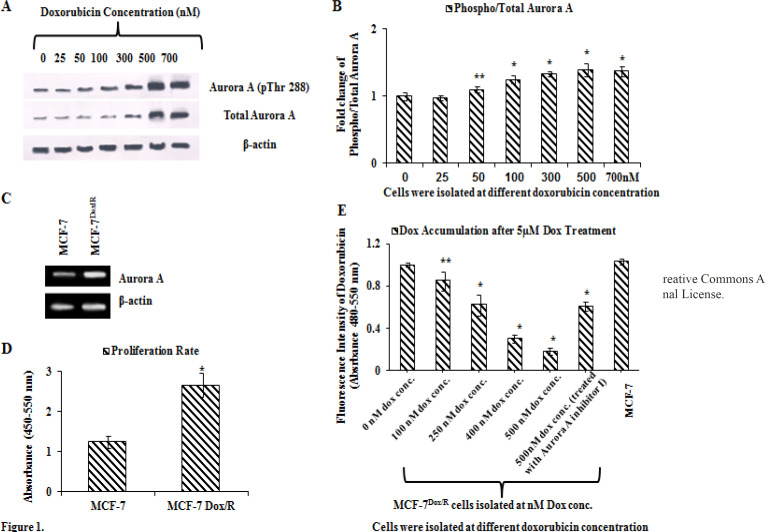
Expression of Aurora-A during incremental Treatment Doses of Doxorubicin (Dox). A. Expressions of Aurora-A (pThr 288) and total Aurora-A were observed during stepwise increasing doses of Dox. At each of the incremental stages proteins were isolated for western blot analysis. β-actin was used as loading control. B. Densitometric analysis & corresponding fold-changes as calculated from the bands obtained by western blot analysis of Aurora-A (pThr 288) and total Aurora-A using ImageJ software. The data was normalized with the β-actin bands. Each bar corresponded to the mean ± SD for three independent experiments. p values **p<0.01 and *p<0.005. C. mRNA levels of Aurora-A were assessed in both the cell lines. Total RNA was isolated and reverse transcribed. The resulting cDNAs were subjected to PCR with primers and the reaction products were electrophoresed and visualized by EtBr staining. β-actin was used as an internal control. D. Measurement of proliferation rate using cell proliferation assay kit. BrdU incorporation within the cells (after incubation with BrdU working solution) was measured by Spectrophotometric plate reader at dual wavelengths of 450-540 nm; *p represents p<0.005. E. MCF-7 Dox/R cells were isolated at different doxorubicin concentration. Spectrofluorimetric measurements of intracellular accumulation of doxorubicin were carried out at each incremental isolated subline. Absorbance was taken at 550 nm. The experiments were repeated thrice. *p represents p<0.005 and **p represents p<0.01 in comparison to MCF-7. BrdU: 5-bromo-2'-deoxyuridine. Dox: Doxorubicin

Involvement of Aurora A in the context of reduced sensitivity towards doxorubicin was initially checked by examining the expression levels of Aurora A at the protein level (phospho Aurora A at Thr 288 and total Aurora A) during administration of incremental doses of doxorubicin in MCF-7 (Figure 1A). Densitometric analysis & corresponding fold-changes of phospho to total Aurora A was calculated from the bands obtained by western blot analysis using ImageJ software. The data was also normalized with the β-actin bands and indicated enhanced fold change with incremental dose of doxorubicin (Figure 1B). An upward trend has also been observed at mRNA expression level in MCF-7Dox/R compared to MCF-7 (Figure 1C). The proliferation rate as measured by BrdU assay revealed that MCF-7Dox/R (isolated at 500 nM) showed higher proliferation rate compared to the parental MCF-7 cells (Figure 1D). These two findings clearly revealed a positive association between Aurora A overexpression and increased rate of proliferation. Spectrofluorimetric data reveals lower accumulation of doxorubicin in MCF-7Dox/R compared to MCF-7 when both cells were treated with equal doses of doxorubicin (5μM). (Figure1E). Interestingly, combinatorial treatment of doxorubicin and Aurora A inhibitor I show higher uptake and accumulation of the drug in MCF-7 as well as in MCF-7Dox/R. This result provides an indication of the contributory role of Aurora A in reducing intracellular doxorubicin accumulation. 

*Expressions of phospho Akt (Ser 473) and phospho IκBα (Ser 32) in MCF-7and MCF-7Dox/R are corollary to Aurora A expression*

**Figure 2:**
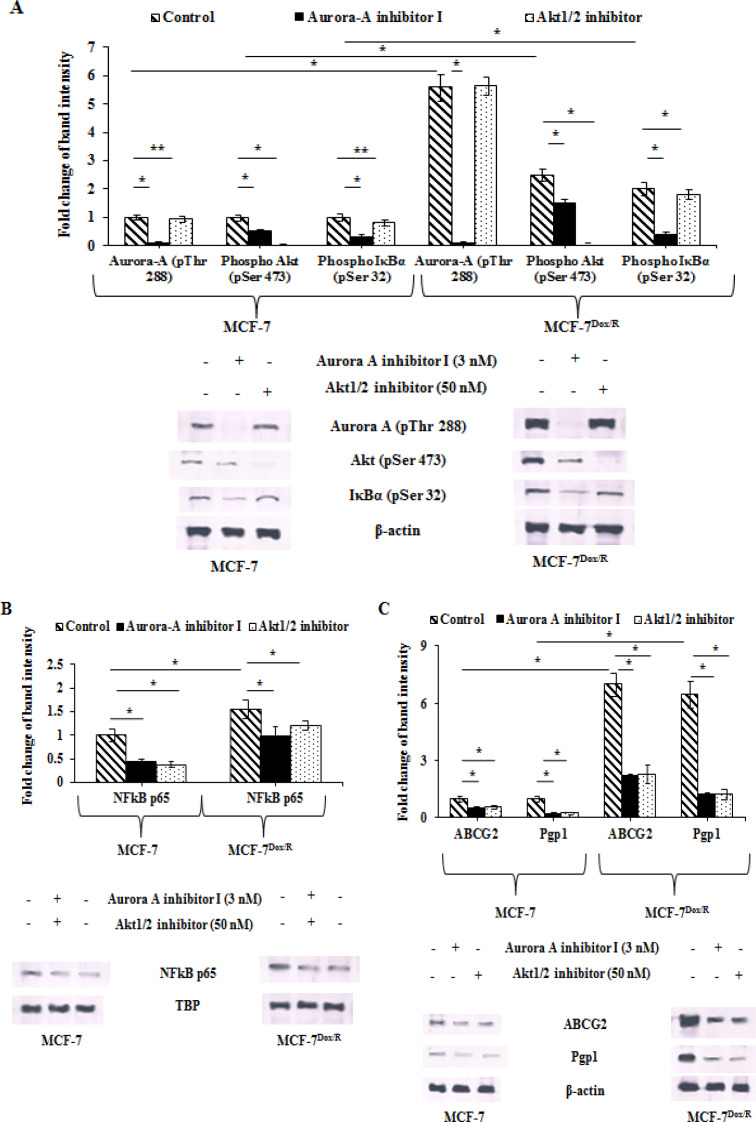
Baseline Expressions and Corresponding Band Intensities of A. Aurora-A (pThr 288, Akt (pSer 473), IκBα (pSer 32), B. NFkB p65, C. ABCG2 and Pgp1 in parental and resistant sublines in presence or absence of either Aurora-A inhibitor I (3nM) or Akt1/2 inhibitor (50nM) for 6h followed by western blot analysis. To ensure equal protein loading β-actin/TBP was used. The experiments were repeated thrice. *p represents p<0.005 and **p represents p<0.01 in comparison to untreated cells. NFkB p65: Nuclear factor k B (p65 subunit); IκBα: nuclear factor of kappa light polypeptide gene enhancer in B-cells inhibitor alpha; ABCG2: ATP-binding cassette super-family G member 2; Pgp1: P-glycoprotein 1; TBP: TATA-Box binding protein

Expressions of phospho-Aurora A (Thr 288) along with two other downstream prosurvival molecules like phospho Akt (Ser 473) and phospho IκBα (Ser 32) were assessed to find out the reason behind increased proliferation and improved survival ability of MCF-7Dox/R . MCF-7Dox/R in the absence of Aurora A inhibitor I exhibited elevated expression of phospho-Aurora A, phospho Akt and phospho IκBα. While these cells treated with Aurora A inhibitor I (3 nM) for 6h, had reduced expression levels Akt and IκBα. On the contrary inhibition of Akt by treatment with Akt1/2 inhibitor (at 50 nM) reduced the expression levels of IκBα, but not Aurora A (Figure 2A). Taken together, these findings manifested a possibility of IκBα, Aurora A and Akt sharing a common signalling pathway where IκBα might perform as a downstream molecule of both Akt and Aurora A. Increased IκBα phosphorylation is a pivotal point for NF-κB p65 signaling pathway (Briassouli et al 2007). 

*Aurora A increases expression of NF-κB*

Inhibitors of Aurora A and Akt kinases were used to observe their effect on NF-κB p65. Result revealed reduced p65 expression in presence of either of the inhibitors (Figure 2B). Overexpression of Aurora A positively correlated with upregulated expression of NF-κB p65 in MCF-7Dox/R. Treatment of Aurora A inhibitor resulted in significantly diminished expression of NF-κB p65 than that found in the cell treated with Akt1/2 inhibitor. These results pointed out the fact of Aurora A mediated NF-κB p65 regulation via Akt pathway. The protein levels of NF-κB p65 in the presence of Aurora A inhibitor I or Akt 1/2 inhibitor was decreased in parental MCF-7 in a differential manner. In all probability, the overexpression of Aurora A in MCF-7Dox/R has influenced the hyperactivation of NF-κB p65 in this resistant subline. These cells when treated with Aurora A inhibitor I and Akt 1/2 inhibitor separately, exhibited lowered expression of NF-κB p65 when compared to untreated cells. Moreover, Aurora A inhibition led to reduced expression of this protein than Akt1/2 inhibitor. This gave an insight to the fact that Aurora A acts superiorly than Akt to activate NF-κB and Akt might be a downstream mediator of NF-κB activation.

*Aurora A upregulates the expression of cell membrane associated efflux pumps*

To explore the probable explanation behind reduced doxorubicin accumulation in MCF-7Dox/R as observed earlier (Figure 1E), expression profiles of cell membrane associated drug efflux pumps were examined. Pgp1 expression was found to be elevated in the absence of Aurora A inhibitor I and was decreased upon suppression of Aurora A by Aurora A inhibitor I in MCF-7Dox/R. We furthermore looked into the expression of ABCG2, a determinant biomarker of chemoresistance. Administration of Aurora A or Akt inhibitor depleted its expression significantly. Diminished expression levels of both Pgp1 and ABCG2 in presence of inhibitors suggested a positive association between drug efflux markers with Aurora A; which may be a direct association or through Akt (Figure 2C). 

*Differential uptake and localization of Curcumin in parental MCF-7 and MCF-7Dox/R*

**Figure 3:**
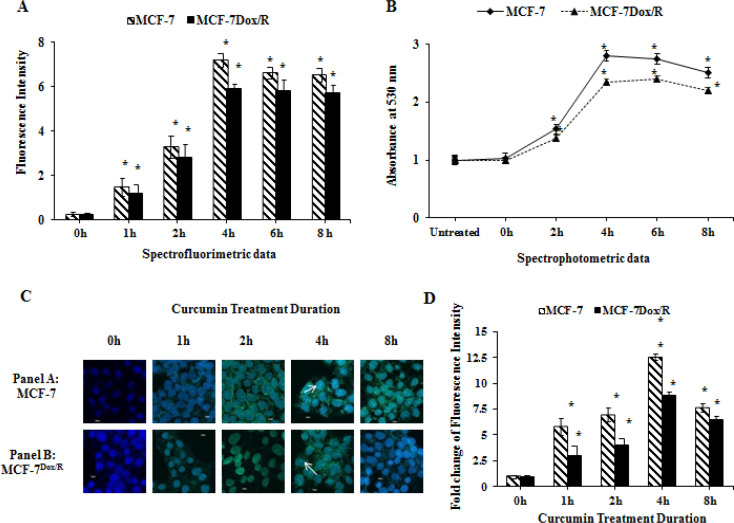
Intracellular Uptake of Curcumin was Studied for Different Time Periods in both MCF-7 and MCF-7Dox/R Cells by A. Spectrofluorimetric measurement and B. spectrophotometric quantitation. Absorbance was taken at 530 nm as excitation emission spectra of curcumin are 420 nm and 530 nm respectively for MCF-7 and MCF-7Dox/R. C. Localization of curcumin (green fluorescence) within the nucleus of both the cells (after treatment for different time periods) was visualized under Confocal Laser Scanning Microscope (60X objective, 2X zoom). DAPI (blue fluorescence) acted as a nuclear stain indicated its co-localization with curcumin (white arrows). D. Based on this Confocal image (Figure C), fold change values of fluorescence intensity was quantitated using ImageJ software and plotted graphically. Values plotted in all the graphs were mean±SD, n=3. *p<0.005 was significant compared to curcumin uptake for 0h. DAPI: 4′,6-diamidino-2-phenylindole

It is important to measure time dependent incorporation of curcumin within the cell. Spectrofluorimetric and spectrophotometric quantitation along with the confocal microscopic imaging of the nuclear localization of curcumin were performed. Fluorescence intensity of curcumin itself indicated that maximum uptake of the phytochemical was achieved within 4h of incubation in both MCF-7 and MCF-7Dox/R cells, although extent of uptake varied with cell types (Figure 3A). Spectrophotometric data reflected the similar trends when absorbance was taken into consideration (Figure 3B). Confocal microscopic data displayed nuclear localization of curcumin in both MCF-7 and MCF-7Dox/R cells after treatment. Filter used for this localization study was based on excitation and emission spectra of curcumin (420 nm and 530 nm respectively). Merged images of curcumin loaded DAPI stained cells (overlapping green fluorescence for curcumin and blue fluorescence for DAPI) presumably indicated (white arrows) the localization of curcumin within the nucleus (Figure 3C). Significant alteration of fluorescence intensity in terms of fold change based on Figure 3C specified maximum uptake of curcumin at around 4 hours of treatment (Figure 3D). Therefore confocal microscopic images correlated with the results obtained from both spectrophotometric and spectrofluorimetric studies. 

*Curcumin rescues doxorubicin sensitivity by reducing EC50 values of doxorubicin in MCF-7 and MCF-7Dox/R*

**Figure 4:**
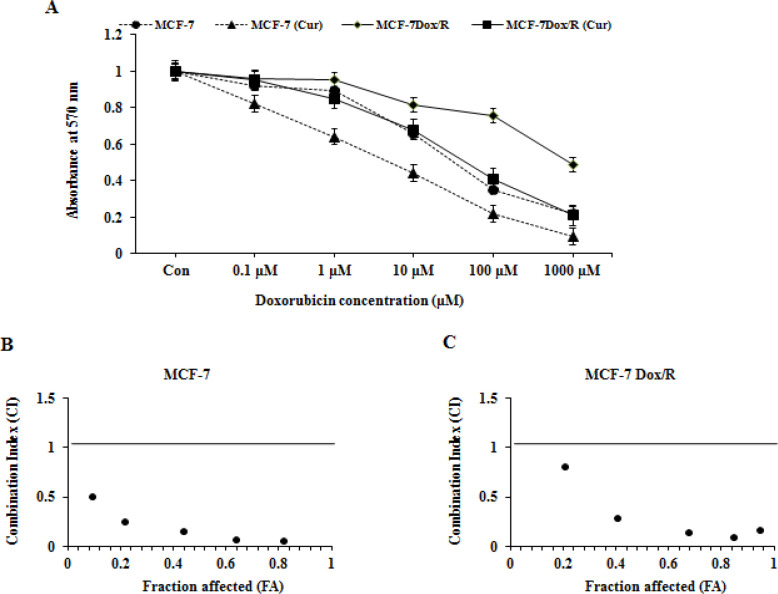
Curcumin as Resistance Modifying Agent. A. Effect of curcumin in reversing acquired resistance towards Dox. Both MCF-7 and MCF-7Dox/R cells were pre-treated with curcumin (25 µM) for 6h followed by Dox treatment for another 24h. Cytotoxicity was measured by MTT reduction assay. The error bars indicated standard deviations of three samples. B and C. Chou-Talalay method of FA-CI plot was adopted to assess synergistic activities of curcumin and Dox in MCF-7 and MCF-7Dox/R. FA-CI: Fraction Affected-Combination Index

Based on intracellular uptake of curcumin, experiment was conducted to determine whether curcumin could reverse the acquired insensitivity induced by doxorubicin. To achieve this goal, both parental and doxorubicin resistant subline were pre-treated with curcumin (25 µM) for 6h followed by treatment with doxorubicin at logarithmic doses for another 24h. From the result (Figure 4A) obtained from MTT assay, EC50 values of doxorubicin were calculated in curcumin pre-incubated and untreated cells. EC50 values of doxorubicin decreased significantly when both the cells were pre-incubated with curcumin (Table 1). The reversal index (RI) = EC50 (chemotherapeutic drug alone) / EC50 (chemotherapeutic drug in presence of curcumin) were calculated (Table 1). The values assertively indicated that curcumin was efficient in imparting sensitivity of resistant cells towards doxorubicin by reversing chemoinsensitivity. For the inspection of synergistic effect between curcumin and doxorubicin in MCF-7 and MCF-7Dox/R cells, combination index (CI) between the two was calculated based on MTT results following the conventional methods (Chou, 2010; Elwakeel et al., 2019). As per graphs plotted, CI values were all less than 1 (Figure 4B and 4C). Mean CI values of both MCF-7 and MCF-7Dox/R cells were 0.199 and 0.291 respectively which were also <1, indicating a strong synergism between curcumin and doxorubicin.

*Curcumin lowers the expression of Aurora A*

**Figure 5:**
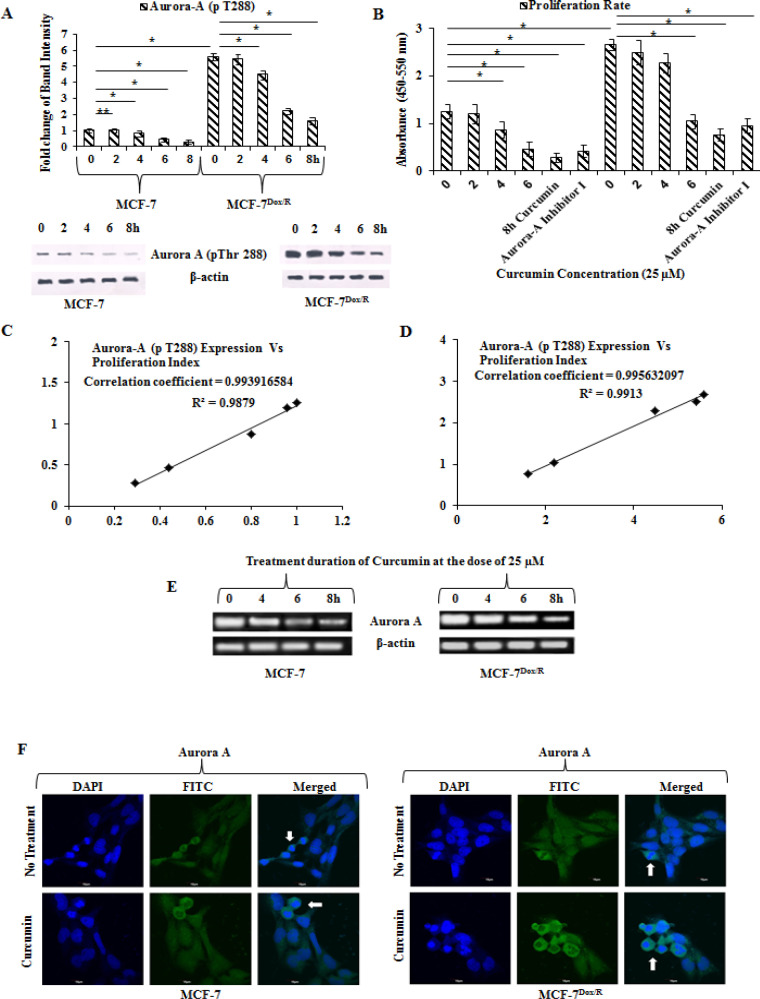
Alteration in Expression Profiles of Aurora-A and Proliferation Rate in Presence of Curcumin. MCF-7 and MCF-7Dox/R cells were treated with curcumin for different time periods. A. Expression levels of Aurora-A (pThr 288) protein and corresponding band intensities after normalisation with β-actin were plotted. B. Proliferation rate in presence or absence of curcumin was quantitated using BrdU cell proliferation assay kit. Experimental result was validated using specific Aurora-A inhibitor. Correlation coefficient and R2 values were measured in Excel using CORREL function and from Microsoft Excel function. C and D. R2 value in MCF-7 and MCF-7Dox/R was closer to 1, indicated better fitness of the trend line. E. RT-PCR technique was followed for expression of mRNA in both the cells following the protocol mentioned earlier. β-actin was used as internal loading control in both the experimental set up. F. Centrosomal localization of Aurora-A in MCF-7 and MCF-7Dox/R cells were indicated with white arrows. Curcumin disrupted Aurora-A localization and induced cell cycle arrest by chromosomal alignment alteration in both th cells. R2: Coefficient of Determination, RT-PCR: Reverse Transcriptase Polymerase Chain Reaction

The previous result gave an insight about effectiveness of curcumin in reversing doxorubicin insensitivity. Since Aurora A is responsible for decreasing doxorubicin accumulation by increasing resistant phenomena, modulatory effect of curcumin over Aurora A was intended to examine. Results indicated a time dependent inhibition of phospho Aurora A in both the cell lines (Figure 5A). Following similar experimental condition, proliferation rate was measured (BrdU assay). Proliferation rate as calculated from the BrdU result, showed a declined trend of rate of proliferation after treatment with curcumin for different time periods. Interestingly, the rate of proliferation of MCF-7Dox/R cells decreased drastically and dropped to almost at the level of parental MCF-7 when treatment duration of curcumin continued for 6 h and beyond. This experiment was validated further using specific Aurora A inhibitor (Aurora A inhibitor I), which clearly conferred that inhibition of Aurora A was associated with decreased proliferation rate. To authenticate the observation of Figure 5A and 5B, we furthermore took help of CORREL function in Excel to find out the correlation coefficient between two variables; Aurora A (pThr288) expression vs proliferation rate. Correlation coefficient values (0.993916584 for MCF-7) and (0.995632097 for MCF-7Dox/R) indicated a perfect positive correlation. Coefficient of Determination R2 was closer to 1 which indicated a better fitness of the trend line. Expression of mRNAs of Aurora A in presence curcumin was found to diminish with time (Figure 5E). To establish the role of Aurora A in mitotic events, immunofluorescence assay was carried out in both parental and resistant sublines in presence and absence of curcumin. Distinct localization of Aurora A was noted in different phases of mitotic events. In absence of curcumin, clear localization of Aurora A near centrosome during metaphase stage was observed (Figure 5F). In curcumin treated cells (MCF-7 and MCF-7Dox/R), on the contrary, a severe arrest was observed due to lack of proper segregation and separation of chromosomes; demonstrating severe arrest of cells at metaphase stage by regulating Auroras (Figure 5F). 

*Curcumin stabilizes p53 by blocking Aurora A*

**Figure 6:**
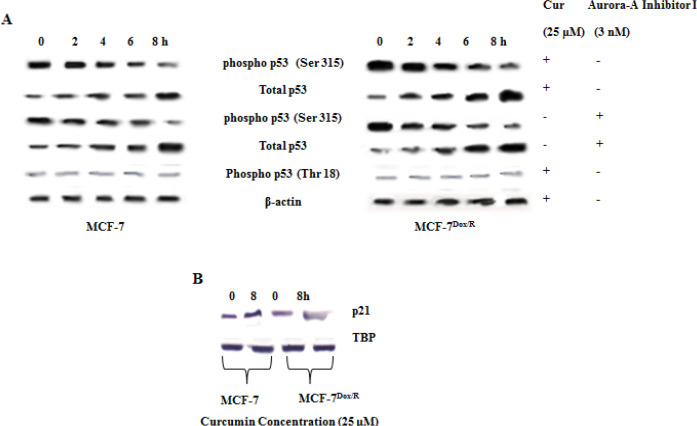
Expressions of Downstream Target Proteins of Aurora-A in Presence and Absence of Curcumin or Specific Inhibitors as Revealed from Western Blot Analysis. A. Expressions of p53 (pSer 315 & pThr 18) residues and total p53 in whole cell lysates were examined following Immunoblotting. Experiment was validated after treatment of cells with Aurora-A Inhibitor I (3 nM). B. Expression of p21 in both the cells. Cur: Curcumin; TBP: TATA-Box binding protein

In order to confirm the contribution of Aurora A in chemoinsensitivity, the expression levels of one of its target molecule p53 was analyzed. The ratio of phospho (ser 315) and total p53 was calculated in both parental and resistant cells (Table 2). The value indicated higher amount of phospho p53 in MCF-7Dox/R compared to parental MCF-7 which might be because of increased amount of Aurora A mediated phosphorylation of p53 in MCF-7Dox/R. Next it was attempted to find out whether curcumin, by virtue of inhibiting Aurora A, could affect its major target protein p53. Untreated cells showed high levels of phospho-p53 at serine 315 residues. Treatment with curcumin reduced phosphorylation status of the protein. The results were analogous to those obtained after treatment with Aurora A inhibitor I (Figure 6A). Status of phospho-p53 (Thr 18), an important phosphorylation site for stability of the protein were examined and contrarily found no alteration in the expression by curcumin (Figure 6A). Expression of cell cycle inhibitory protein p21 was upregulated considerably by curcumin (Figure 6B). These results cumulatively focussed on the efficacy of curcumin in targeting Aurora A and its decisive downstream target proteins. 

*Curcumin synergizes doxorubicin to induce G2/M arrest and subsequent apoptosis*

**Figure 7:**
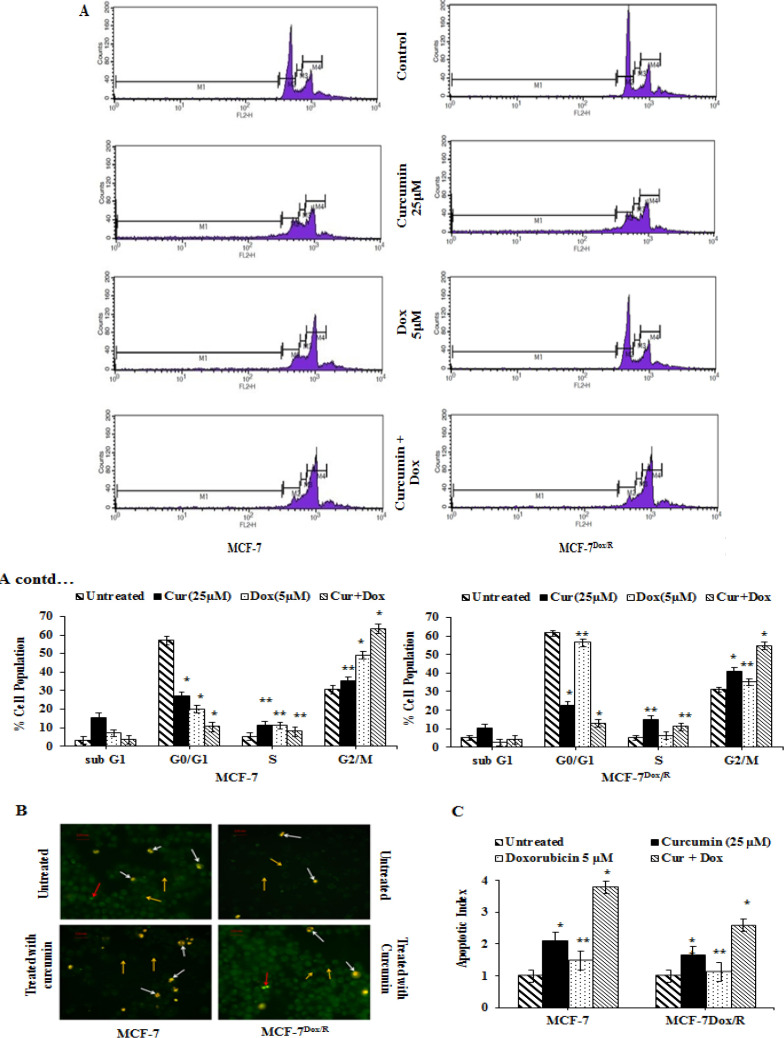
Analysis of cell cycle distribution by Flow Cytometry after staining with PI and apoptotic cells by Annexin V- FITC/ PI staining. A. MCF-7 and MCF-7Dox/R cells were treated with either curcumin (6h) or doxorubicin (12h) or pretreatment with curcumin (6h) followed by Dox treatment (12h). Indication of significant G2/M arrest was observed in both the cells when treated with curcumin along with doxorubicin. Distribution of cells in different stages of cell cycle was observed from histogram results. The results were representative of three independent experiments. Values represented mean ± S.D (n=3). *p<0.005 and **p<0.01 were significantly different compared with untreated counterparts. B. Detection of apoptotic and non-apoptotic cells following Annexin V- FITC/ PI staining after treatment of MCF-7 and MCF-7Dox/R cells with curcumin (25 μM). Cells were visualized under fluorescence microscopy at 20X magnification. Annexin V single positive cells (bright green) were early apoptotic as indicated by red arrow, double positive cells (yellowish orange) were late apoptotic as indicated by white arrow, control double negative cells (very faint green) were non-apoptotic healthy cells, indicated by yellow arrow. C. Calculation and graphical representation of apoptotic indices for MCF-7 and MCF-7Dox/R cells treated with either curcumin or doxorubicin or curcumin pre-treatment followed by doxorubicin treatment as mentioned in the Result section. Each treatment was scored for 100 cells at random under fluorescent microscope and classified into apoptotic and non-apoptotic cells based on their characteristic features. The ratio of apoptotic to non-apoptotic cells was designated as apoptotic index. Values represented mean ± SE (n=100). Values were significant *p<0.005, **p<0.01 with respect to untreated control. Cur: Curcumin; Dox: Doxorubicin; FITC: Fluorescein-5-isothiocyanate; PI: Propidium Iodide

Next it was examined whether curcumin exerted any influence over doxorubicin induced growth arrest. In MCF-7 cells, as obvious from the results, doxorubicin alone induced G2/M arrest and number of cell population at G2/M phase was furthermore outstretched by curcumin when cells were treated with same concentration of curcumin as that was used for regulating Aurora A (Figure 7A). Resistant subline (MCF-7Dox/R) instead failed to exert G2/M arrest in presence of the drug, further confirming its insensitivity. However, in case of combination treatment where cells were pre-treated with curcumin (25 μM, 6h) followed by doxorubicin treatment for another 12h, significant G2/M arrest was observed in resistant subline too (Figure 7A). MCF-7 and MCF-7Dox/R cells when treated with curcumin alone for 12 h was found to induce apoptosis as visualized under fluorescence microscope (Figure 7B). Cells were stained for fluorescence microscopy and visualized at 20X magnification. Annexin V single positive cells (bright green) were early apoptotic as indicated by red arrow, double positive cells (yellowish orange) were late apoptotic as indicated by white arrow, control double negative cells (very faint green) were non-apoptotic healthy cells, indicated by yellow arrow. Curcumin treated MCF-7 cells underwent apoptosis as observed in the figure compared to the untreated MCF-7 cells. Conversely, very few MCF-7Dox/R cells were directed to the programmed cell death when cells were treated with curcumin. The result was in support with the notion that curcumin was inducing apoptosis in resistant subline too, although extent was differential. In a different set of experiment both the sublines were treated with either curcumin alone (12 h) or doxorubicin (12 h) or pretreatment with curcumin followed by doxorubicin treatment. Cells were observed under microscope and almost 100 cells were counted from each slide and designated into apoptotic and non-apoptotic, based on characteristic morphological features. Apoptotic index (ratio of apoptotic to non apoptotic cells) was calculated and was found to be much higher in case of cells pretreated with curcumin prior to doxorubicin treatment (microscopic image not given). The result was displayed in Figure 7C.

## Discussion

The major objective of this study was to determine whether Aurora A is responsible for reduced sensitivity towards doxorubicin in MCF-7Dox/R and the role of natural phytochemical curcumin in rescuing doxorubicin responsiveness has also been elucidated. To address the problem, the expression levels of Aurora A in parental MCF-7 and in MCF-7Dox/R isolated at different doses of treatment were explored. Results indicated that Aurora A got overexpressed in the resistant subline. Additionally, observation revealed that particularly the phosphorylated (Thr 288) form of Aurora A expression was uplifted, a marker of functionally activated form of Aurora A. Cells isolated at 500 nM (MCF-7Dox/R) exhibited highest expression of the enzyme. These observations were further supported by the increased expression of mRNA level of Aurora A in MCF-7Dox/R cells as revealed in the result; implying the probable activation of Aurora A at both transcriptional and translational levels to accomplish doxorubicin insensitivity in MCF-7Dox/R. Increased proliferation rate in MCF-7Dox/R cells furthermore warranted acquired insensitivity towards the drug. Decreasing tendency towards intracellular accumulation of doxorubicin was evident with gradual shifting from parental MCF-7 towards drug resistant subline. Surprisingly, administration of Aurora A inhibitor aids in drug accumulation and thus ascertaining the involvement of Aurora A in obstructing intracellular drug accumulation. 

To provide an insight into the underlying mechanism of Aurora A mediated doxorubicin insensitivity, expression profiles of phospho Akt, phospho IκBα and NFκB p65 were assessed. Expressions of phospho Akt and phospho IκBα were significantly higher in MCF-7Dox/R cells. However, inhibition of Aurora A by administration of Aurora A inhibitor I led to concomitant depletion of phospho Akt and phospho IκBα. These findings are in agreement with those of recent studies where Aurora A was found to phosphorylate IκBα at ser 32 and Akt at ser 473 residues respectively (Briassouli et al., 2007; Wu et al., 2019; Biswas et al., 2020). ). Our findings also established higher expression of p65 as a consequence of IκBα phosphorylation by Aurora A. These findings gave rise to a possibility of an involvement of Aurora A-Akt–NFκB signaling axis in the development of doxorubicin insensitivity. Previous studies reported that Aurora A activates Akt by phosphrylating its ser 473 residue. Phospho Akt in turn phosphorylates IKK (IκB kinase) and eventually facilitates nuclear translocation of NFκB p65; (Huang et al., 2012). NFκB p65 ultimately accelerates the transcriptional rate of Pgp1 (Kim et al., 2011; Li et al., 2018). Accordingly in our present findings the involvement of the above mentioned signalling pathway showed its contributory effect to lower the efficacy of doxorubicin in MCF-7Dox/R. Additionally, we extended the scope of these findings by observing the expression profiles of Pgp1 and ABCG2, two important signature markers of chemoinsensitivity. Considerably higher expressions of both the drug efflux markers as observed in MCF-7Dox/R cells were downregulated upon treatment of cells with Aurora A inhibitor I as well as Akt1/2 inhibitor. Results obtained clearly signified Aurora A mediated upregulation of drug efflux pumps through Akt-NFκB. Growing evidences establishing a link between Akt activation and ABCG2 overexpression (Huang et al., 2014; Hu et al., 2016; Wang et al., 2019), also supported the present findings. Overall data trails to this conclusion that a cascade of functional activation of Aurora A-Akt- NFκB cumulatively triggers drug efflux pumps like Pgp1and ABCG2 and causes higher expulsion of doxorubicin in MCF-7Dox/R culminating in poor response towards doxorubicin treatment. 

Curcumin, the principal component of turmeric has been established as a potent anticancer agent due to its wide spectrum of biological activities. Compelling evidences indicated involvement of curcumin in reversing insensitivity towards chemotherapy by regulating several molecular pathways (Sen et al., 2011; Jiang et al., 2013; Thulasiraman et al., 2014). These studies encouraged us to apply curcumin as a chemoenhancer molecule in our present study. Initially MCF-7 and MCF-7Dox/R cells were treated with curcumin to find out its IC50 values (data not given) in both cells. We also confirmed the uptake of curcumin in both of the cells by measuring the fluorescence intensity of curcumin in spectrofluorimeter and detecting optical density in spectrophotometer. These results strongly correlated with the confocal microscopic images of intracellular localization of curcumin in parental MCF-7 and MCF-7Dox/R. Subsequently both cells were pretreated with curcumin (25µM for 6 hours) followed by treatment with doxorubicin. The striking reduction of EC50 value of doxorubicin in curcumin pretreated cells compared to untreated cells conferred the effectiveness of doxorubicin in presence of curcumin in both MCF-7 and MCF-7Dox/R. Curcumin reduced the resistance towards doxorubicin by 7.25 fold in MCF-7Dox/R as calculated from reversal index. (Table 1) Combination Index value exhibited a strong synergism between curcumin and doxorubicin. Gradual decrease in the OD value in curcumin preteated samples showed its effect in improving doxorubicin induced cell killing not only in parental MCF-7 but also in MCF-7Dox/R; thus reversing the doxorubicin insensitivity in resistant subline. 

Results obtained so far have showed that curcumin was able to rescue the effectiveness of doxorubicin. Since Aurora A overexpression is correlated with reduced doxorubicin sensitivity, next attempt was taken to find any relationship between alteration in expression of Aurora A and increased curcumin mediated efficacy of doxorubicin. . The expression of Aurora A at mRNA level was decreased gradually up to the incremental treatment duration of curcumin for 8 hours; providing a clue of curcumin mediated transcriptional control of Aurora A. Consequently total Aurora A in protein level showed decreased expression upon treatment with curcumin. (Figure 5) Diminished phospho-Aurora A expression accompanied by decreased proliferation rate as observed after curcumin treatment established a strong positive association between phospho Aurora A expression and cellular proliferation (Figure-5). This was further verified when the doxorubicin efficacy was improved in terms of reduced proliferative rate of cells in samples after treatment with Aurora A inhibitor I as well as in cells pretreated with curcumin. Moreover curcumin treated cells showed inappropriate localization of Aurora A (Immunofluorescence assay) instead of its usual location at centrosome which might inhibit Aurora A to carry on its mitotic activity in cells. Taken together curcumin by lowering the expression and functional activation of Aurora A allows doxorubicin mediated killing in cancer cells which justified the use of this phytochemical in as a chemoenhancer molecule.

Several reports explained an interaction of Aurora A with p53, as the enzyme phosphorylates p53 at serine 215 and serine 315 residues and ultimately inactivates the protein and facilitates oncogenic transformation of cells by activating the prosurvival pathways via several mitotic and non-mitotic mechanisms (Liu et al., 2011; Hsueh et al., 2013; Sasai et al., 2016). Expression of phospho-p53 (Ser315) in MCF-7Dox/R was higher than MCF-7 (Figure 6A). Upregulated phosphor p53, which ultimately leads to proteasomal degradation, might be one of the explanations behind reduced rate of doxorubicin induced apoptosis in MCF-7Dox/R and thus causing insensitivity of cells towards the drug. Another fascinating finding of this study was that curcumin by repressing Aurora A simultaneously depleted phospho-p53 (Ser 315) vis-a-vis elevated expression of p21 (downstream molecule of p53). Total p53 instead, was found to be upregulated with treatment duration of curcumin. Curcumin instigated the reduction of phospho-p53 in MCF-7 as well as in MCF-7Dox/R and activated p53. This activation might be linked with a number of possibilities ranging from p53 mediated transcriptional control of Aurora A (Wu et al, 2012) and p53 stimulated apoptosis in resistant subline. Thus, curcumin reversed doxorubicin insensitivity in a multifaceted approach by blocking Aurora A either by its own involvement in the transcriptional silencing of Aurora A or allowing p53 mediated regulation of Aurora A expression and subsequent stimulation of apoptosis. 

Next objective of the study was to ensure whether curcumin by regulating Aurora A could aid in doxorubicin mediated growth arrest and ultimately apoptosis and thus reverses insensitivity. Pre-treatment of cells with curcumin followed by doxorubicin treatment efficiently induced cell cycle arrest at G2/M phase as observed by Flow Cytometry. Previous studies have also reported that other compounds in combination with curcumin imparted better efficacy in inducing cell cycle arrest (Patel et al., 2015; Huang et al., 2017). Curcumin driven apoptosis in cancer cells have been reported by other researchers including that of our laboratory (Wu et al., 2010; Mukherjee et al., 2012; Lv et al., 2014). Furthermore researchers observed the efficacious role of curcumin to improve sensity of tumor cells towards chemotherapeytivc drugs like doxorubicin and 5-Fluorouracil (Khameneh et al., 2019; Sarkhosh et al ., 2018). Present findings are also in agreement with those of others. It was observed when curcumin pre-treated cells were subjected to doxorubicin treatment, frequency of apoptotic cells were much higher which was reflected in calculating apoptotic index values as plotted in the graph mentioned in the result section. In both the experimental set up of growth arrest and apoptosis, combination treatment gave rise to better impact on both MCF-7 and MCF-7Dox/R. Taken together the present findings established the role of curcumin in reversing doxorubicin insensitivity and increasing sensitivity by targeting Aurora A and suggestively attributed the role of curcumin in accelerating cell cycle arrest and apoptosis induced by doxorubicin.

**Table 1:** Reversal of Acquired Resistance in Breast Cancer Cells by Curcumin

Compound	MCF-7		MCF-7Dox/R	
	EC_50_ Dox (μM)	RI	EC_50_ Dox (μM)	RI
Compound (-)	85±2.2	-	870±5.2	-
Cur (25 μM)	5±0.4*	17	120±3.2*	7.25

Values are mean ± SD, (n = 3); RI, Reversal Index; *p <.005 vs. untreated; RI, EC_50_ (chemotherapeutic drug alone) / EC_50_ (chemotherapeutic drug in presence of curcumin)

**Table 2:** Comparative Band Intensities of Phospho as Well as Total p53 Proteins as Obtained from Western Blot Analysis in Presence of Either Curcumin or Aurora-A Inhibitor I

	MCF-7		MCF-7Dox/R	
Treatment Duration	Phospho p53/Total p53	Phospho p53/Total p53	Phospho p53/Total p53	Phospho p53/Total p53
	(Treatment with Curcumin at 25 µM)	(Treatment with Aurora A inhibitor I at 3 nM)	(Curcumin Treatment at 25 µM)	(Treatment with Aurora A inhibitor I at 3 nM)
0h	3.48±0.5	3.86±0.4	5.45±0.5	5.12±0.4
4h	1.3±0.32	1.63±0.2	2.6±0.32	2.4±0.2
8h	0.82±0.05	0.98±0.04	1.32±0.09	1.15±0.04
12h	0.42±0.03	0.62±0.02	0.6±0.05	0.39±0.02
16	0.18±0.03	0.15±0.02	0.35±0.03	0.24±0.02

## Author Contribution Statement

Souvick Biswas: Execution of entire research work, analysis of data and writing of the article. Elizabeth Mahapatra: Assisted with Data analysis and literature survey. Archismaan Ghosh: Assisted with Data acquirement, literature survey. Salini Das: Assisted with Data analysis and literature survey. Madhumita Roy: Guidance, and final approval of the manuscript. Sutapa Mukherjee: Guidance, assisted with data analysis, interpretation and manuscript checking and final approval of the manuscript.

## Part of Student’s Thesis

The study has been approved by the Department of Biotechnology, University of Calcutta as a part of Ph.D thesis work of Mr Souvick Biswas.
